# Telomere shortening and mitotic dysfunction generate cytogenetic heterogeneity in a subgroup of renal cell carcinomas

**DOI:** 10.1038/sj.bjc.6601803

**Published:** 2004-05-25

**Authors:** D Gisselsson, L Gorunova, M Höglund, N Mandahl, P Elfving

**Affiliations:** 1 Department of Clinical Genetics, University Hospital, Lund SE-221 85, Sweden; 2 Department of Urology, University Hospital, Lund SE-221 85, Sweden

**Keywords:** renal cell carcinoma, chromosome instability, telomere, breakage–fusion–bridge cycle, centrosome

## Abstract

Most renal cell carcinomas (RCC) show only simple chromosomal changes. However, a more complex cytogenetic pattern has been found in a subgroup of aggressive RCC, indicating that further accumulation of chromosome changes could play a role in tumour progression. To explore the possible mechanisms behind cytogenetic evolution in RCC, a parallel assessment of chromosome mutations and mitotic segregation pattern in eight tumours was performed. In the majority of cases, no abnormalities in the cell division machinery were found and the rate of alterations in chromosome copy number, as measured by interphase FISH, was similar to that in non-neoplastic cells. This was reflected by relatively simple karyotypes, with little cytogenetic intratumour heterogeneity. In contrast, another group of tumours exhibited several cytogenetically related clones with additional structural chromosomal changes at two or more ploidy levels and a frequency of copy number alterations that was higher than in normal cells. In these cases, the telomere repeat sequences were abnormally short and chromosomal breakage–fusion–bridge events were observed at cell division, as well as multipolar configurations and supernumerary centrosomes. Abnormalities of the cell division machinery may thus contribute to the evolution of complex karyotypes and genetic intratumour heterogeneity in a subgroup of RCC.

Renal cell carcinomas (RCC) typically exhibit relatively simple chromosome changes, and a strong correlation has been established between the cytogenetic abnormalities and the histopathological classification (Kovacs *et al*, 1997). Unbalanced structural rearrangements, leading to deletions in the short arm of chromosome 3, are common in nonpapillary (clear cell) RCC, but are rare in papillary tumours. Papillary RCC, on the other hand, often exhibit polysomies for chromosomes 7, 12 and 17, and loss of the Y chromosome. Little evidence of genetic intratumour heterogeneity has been presented in RCC: of the 818 published and cytogenetically investigated cases of renal adenocarcinoma, only 193 cases (24%) showed more than one clone, and only 71 cases (9%) had more than two clones (Mitelman *et al*, 2003). This is in sharp contrast to the scenario in many other epithelial malignancies, which often exhibit highly complex cytogenetic patterns. Furthermore, the TP53 protein, which is believed to be an important safeguard against DNA damage and is inactivated in the majority of human cancers, is only inactivated in approximately 20% of RCC (Contractor *et al*, 1997). Nonetheless, *TP53* mutation as well as aneuploidy have been suggested to predict poor survival among these patients (Ljungberg *et al*, 1996; Girgin *et al*, 1999), indicating that some tumours may acquire a more aggressive phenotype through the disruption of cell cycle checkpoints and subsequent cytogenetic evolution.

One mechanism that may induce complex chromosome rearrangements is telomere shortening. When the number of TTAGGG repeats at the chromosome ends reaches a certain critical level, the protective telomeric DNA–protein complex is disrupted (de Lange, 2002). The exposure of free double-stranded DNA then leads to the formation of unstable ring and dicentric chromosomes, which may trigger cytogenetic rearrangements through breakage–fusion–bridge (BFB) events (McClintock, 1940; Gisselsson *et al*, 2001). In RCC, measurements of terminal restriction fragment lengths have demonstrated that 70% of the tumours have shorter telomeres than normal renal epithelial cells (Dahse *et al*, 1999), and a correlation has been observed between pronounced telomere shortening and chromosomal end fusions (Holzman *et al*, 1993). However, whether these processes actually lead to an increased rate of chromosome mutation in RCC has not been assessed. In the present study, telomere status was evaluated in eight RCC and compared to the pattern of cytogenetic changes, the occurrence of cell division abnormalities and the histopathological classification.

## MATERIAL AND METHODS

### Cell culture and chromosome banding

Eight RCC were selected for analysis from a series of consecutive cases based on the finding of an abnormal karyotype: karyotypically normal cases and cases showing only non-clonal aberrations were excluded from the study, whereas all cases with clonal changes were included (Table 1

**Table 1:** Clinical data and karyotypes

**Case**	**Age/Sex^a^**	**Histological type**	**Grade**	**T-stage**	**Karyotype**
1	51/F	Clear cell	I	pT2	45,XX,del(3)(p12),der(13;14)(q10;q10) [15]
2	64/F	Clear cell	I	pT2	45,XX,−3,der(7)t(3;7)(q11;q11),der(19)t(7;19)(q11;q13) [21]/46,XX [22]
3	82/F	Clear cell	I	pT1	42,XX,der(2)t(2;3)(p25;q11),−3,−4,+5,del(11)(p13),−14,der(17)t(17;18)(p11;q11),−18,−19 [5]/46,XX [9]
4	58/F	Clear cell	II	pT3c	47,XX,+3 [cp3]/47–48,X,−X,+3,+17,+r [cp3]/47–49,XX,+17 [2]/47,XX,+7 [cp5]/47,XX,+18 [cp3]/48,XX,+X [cp2]/46,XX [25]
5	79/M	Clear cell	II	pT2N1	47–48,X,−Y,+5,+8,+12,+16,+der(?17)del(17)(p13)del(17)(q23),−18,−20 [cp12]
6	70/M	Clear cell	II	pT1	43,X,−Y,t(1;17)(p36;q21),der(1)t(1;17)(p36;q21),+5,der(?7)t(3;7)(q21;p22),−14,−15,der(15)t(5;15)(q11;p11), dic(16;?)(p13;?),−18 [cp28]/42–43,idem,add(14)(q32),−der(15)t(5;15),+15[cp4]/43,idem,add(14)(q32) [2]/43, idem,dic(14;?)(q32;?)[cp6]/43–44,idem,+r [cp3]/80–86,idemx2 [cp7]/84–86,idemx2,dic(14;?)x2 [cp3]
7	66/M	Clear cell	II	pT3a	44–46,X,−Y,der(1)t(1;1)(p35;q12),+3,der(3)del(3)(p12p21)del(3)(p25)x2,−6,+7,add(11)(q21),−14,−19,−22,+r, +mar [cp20]/43,X,−Y,del(1)(p34),der(3)del(3)del(3),+7,−9,add(11),−14 [cp4]/44,X,−Y,der(3)del(3)del(3),−6,+7, i(8)(q10),t(10;19)(q22;q13),−14 [cp4]/68–71,XX,−Y,+3,der(3)del(3)del(3)x2,−6,+7,+7,−8,+12,−13,−14,der(14)
					t(4;14)(q12;p11),der(14)t(8;14)(q11;p11),+16,−18,+20,+21,+22 [cp18]/83–91,idemx2 [cp13]
8A	39/M	Papillary	II	pT3a	48–49,X,−Y,+7,+7,+12,+17 [25]
8B					48–49,X,−Y,+7,+7,+12,+17 [cp59]/49,idem,der(19)t(3;19)(q13;q13) [cp25]/48,idem,−8,der(15)t(8;15)(q11;p11), der(19)t(3;19) [cp3]/97–98,idemx2,add(19)(q13)x2 [cp3]/46,XY

a F=female; M=male.

). Tumour biopsies were minced with scissors and disaggregated overnight in 180 U ml^−1^ collagenase II (Cooper Biomedical, Lakewood, NJ, USA). The resulting cell suspension was frozen in culture medium with 10% DMSO, and stored in liquid nitrogen until further analysis. After thawing, cells were cultured on chamber slides or in culture flasks in RPMI 1640 medium with HEPES buffer, supplemented with 17% foetal bovine serum, 1 IU ml^−1^ insulin, 1 ng ml^−1^ epidermal growth factor, 0.23 mg ml^−1^ L-glutamine, 100 IU ml^−1^ penicillin and 0.2 mg ml^−1^ streptomycin. After a first harvest and chromosome banding analysis, the remaining cultures were propagated until 90% confluence was reached (3–5 days) and then subcultured once or twice before further analyses. Harvest and chromosome banding with Wright's stain were according to standard methods (Mandahl, 2001).

### Interphase analysis

Interphase fluorescence *in situ* hybridisation (FISH) analysis was performed using commercially available probes for the centromeric alphoid DNA of chromosomes 3, 7, 12, 13 and 17. Stringency washing was in 1 × SSC at 72°C for 2 min and probe detection was according to standard procedures. To assess the degree of variation in chromosome copy number, the proportion of cells with copy numbers outside the modal number was evaluated (Lengauer *et al*, 1997); in cases where karyotypically normal cells were also present in the cultures, the disomic cells were included in the stem line. At least 250 nuclei were analysed for each hybridisation. As controls, karyotypically normal peripheral blood lymphocytes stimulated by phytohaemagglutinin were used. As it could not be excluded that copy-number alterations not present *in vivo* were acquired during prolonged tissue culturing, normal dermal fibroblasts, cultured for four population doublings, were included as an additional reference.

### Analysis of telomeric repeat sequences

Previous studies have shown that a subset of chromosomes with abnormally short or absent telomeric TTAGGG repeats is a more common source of genomic instability in tumours, than overall telomere shortening (Artandi *et al*, 2000; Gisselsson *et al*, 2001). To detect critically short telomeres in individual chromosomes, TTAGGG repeats were visualised by FISH with fluorescein-conjugated (CCCTAA)_3_ peptide nucleic acid probes (Landsdorp *et al*, 1996). Signal intensity was directly quantified by the Cytovision software (Applied Imaging, Newcastle, UK) and the number of negative chromosome termini for each metaphase cell was scored. At least 20 cells were evaluated in each case. This method does not constitute a precise measurement of telomere length, nor does the absence of signal exclude the presence of a low number of remaining TTAGGG repeats. Nonetheless, previous studies have demonstrated that the method yields a valid assessment of the protective capacity of individual telomeres (Gisselsson *et al*, 2001).

### Analysis of mitotic cell morphology

For analysis of mitotic figures, cells on chamber slides were briefly washed in phosphate-buffered saline (PBS), fixed in methanol : acetic acid (3 : 1) at −20°C for 30 min, air-dried, and stained with haematoxylin and eosin. At least 30 anaphase and 100 metaphase cells were analysed in each case.

### Centrosome detection

Cells on chamber slides were washed in PBS for 5 min, fixed in methanol at −20°C for 30 min and air-dried. Centrosomes were then detected as previously described (Gisselsson *et al*, 2002), using murine monoclonal anti-*γ*-tubulin antibodies (GTU-88, Sigma, St. Louis, MS), biotinylated anti-mouse antibodies (E0354, DAKO A/S, Denmark) and streptavidin-Alexa 594 (Molecular Probes, Leiden, the Netherlands). As background, *β*-tubulin was detected by murine monoclonal antibodies (2-28-33, Sigma), followed by anti-mouse antibodies coupled to fluorescein isothiocyanate (F0232, DAKO A/S). In each case, at least 50 cells were evaluated regarding the number and structure of centrosomes. In control fibroblasts, enlarged centrosomes, that is, those containing >2 centrioles, were found in approximately 2% of the cells, whereas no cells with >2 centrosomes were found.

## RESULTS

**Figure 1:**
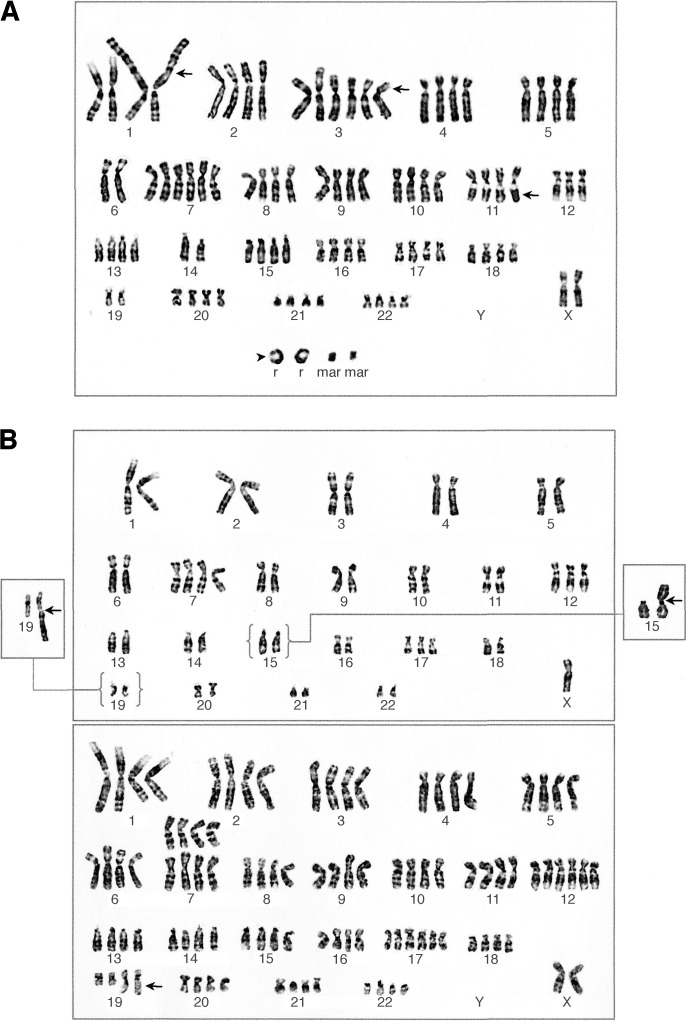
Representative karyotype of case 7, showing several structural changes (arrows) and ring chromosomes (arrowhead; **A**); clonal evolution of structural changes in tumour 8B illustrated by the stem line karyotype (top), identical to that of tumour 8A, the partial karyotypes of the diploid side lines with der(15)t(8;15) (right) and der(19)t(3;19) (left), and the hypertetraploid side line with add(19) (bottom; **B**).

Chromosome banding analysis of cells from primary cultures revealed only relatively simple karyotypes in cases 1–5, whereas cases 6 and 7 showed complex cytogenetic patterns (Table 1). In case 8, two different biopsies from the tumour parenchyma were analysed separately, of which one (A) showed cells with only numerical changes, whereas the other (B) exhibited additional structural aberrations. Of the tumours with simple karyotypes, 1–3, 5 and 8A were monoclonal. Tumour 4 exhibited intratumour variability with regard to numerical aberrations and a ring chromosome, with three related and three unrelated clones. The three cases with complex karyotypes (6, 7 and 8B) all exhibited several related clones with variability in both structural and numerical aberrations (Figure 1

).

**Figure 2:**
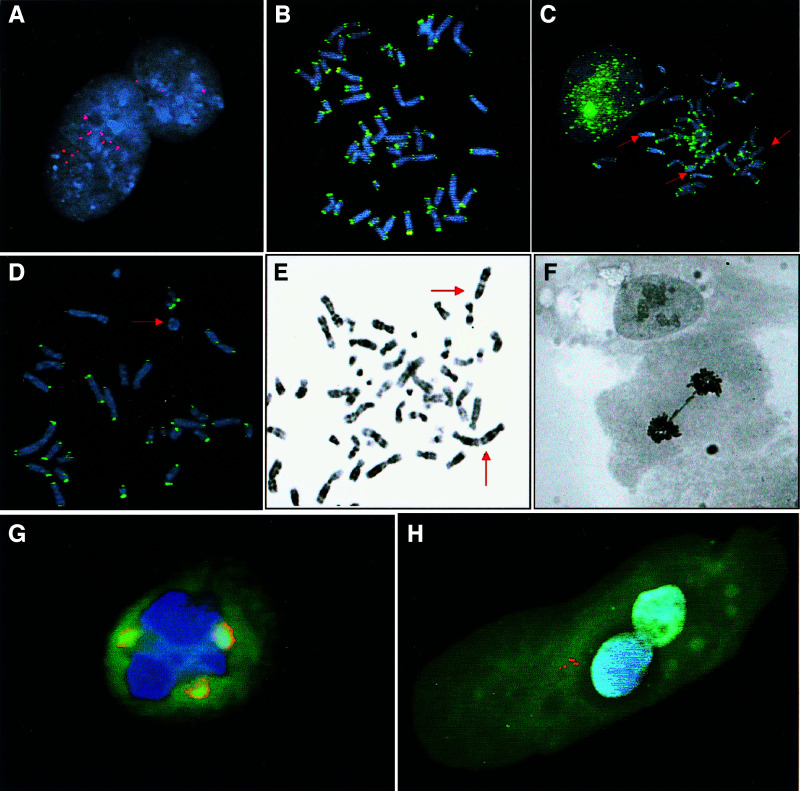
Interphase cell from case 6, showing multiple centromeric signals for chromosome 17 (**A**); normal telomeric signal pattern in case 8A (**B**), compared to 8B (**C**), showing TTAGGG-negative chromosome ends (arrows); multiple chromosome ends and a ring chromosome (arrow) without TTAGGG signals in case 7 (**D**); telomeric fusions (**E**), an anaphase bridge (**F**) and a tripolar cell division coordinated by three centrosomes (orange, **G**) in case 6; multiple centrosomes in a binucleated interphase cell in case 7 (**H**).

To assess the intratumour heterogeneity of numerical chromosome aberrations also in the non-dividing cell population, interphase FISH with centromeric probes for five different chromosomes was performed on cells from six of the tumours (Figure 2A

; Table 2

**Table 2:** Percentage of cells with chromosome copy numbers deviating from the modal value

**Case**	**cen^b^3**	**cen 7**	**cen 12**	**cen 13**	**cen 17**	**Median**
1	5 (2)	5 (2)	4 (2)	6 (2)	5 (2)	5
4	6 (2)	12 (2)	12 (2)	13 (2)	5 (2)	12
5	15 (2)	8 (2)	16 (3)	6 (2)	8 (2)	8
6	21 (2)	23 (2)	26 (2)	22 (2)	19 (2)	22
7	39 (2)	47 (2)	31 (2)	23 (2)	19 (2)	31
8A	2 (2)	26 (4)	2 (2)	1 (2)	8 (3)	2
Fibroblasts	8 (2)	12 (2)	10 (2)	10 (2)	6 (2)	10
Lymphocytes	1 (2)	5 (2)	1 (2)	2 (2)	2 (2)	2

a Modal copy numbes are within parentheses;

b centromeric alpha-satellite probe.

), cultured for approximately four generations (passage 2). All cultures were initiated from approximately the same number of cells. The cases with simple karyotypes (1, 4, 5 and 8A) showed an elevated proportion of cells (>3%) with copy numbers outside the modal number compared to peripheral blood lymphocytes. However, compared to normal dermal fibroblasts cultured for an equal number of generations as the RCC cells, the proportion of cells outside the modal number was either lower or similar. On the other hand, the two cases with complex karyotypes (6 and 7) exhibited rates two and three times higher than the fibroblasts, respectively. The fibroblasts exhibited a normal 46,XY karyotype at both generation 1 and generation 4, although nonclonal changes were present.

When FISH analysis with probes for terminal TTAGGG repeats was performed on all cases, the tumours with simple karyotypes (1–5, and 8A) showed a hybridisation pattern similar to normal lymphocytes and fibroblasts, with a maximum of two chromosome ends below the detection level. The tumours with complex karyotypes (6, 7 and 8B), on the other hand, exhibited a subpopulation of cells with an elevated number of TTAGGG-negative ends (Table 3

**Table 3:** Ploidy levels and cell division abnormalities

**Case**	**Ploidy levels**	**No. of abnormal clones**	**Range of TTAGGG-negative termini^a^**	**Dic/tas/r^b^**	**Anaphase bridges**	**Multipolar mitoses**
1	2n	1	0–2	0	0	0
2	2n	1	0–1	0	1.8%	0
3	2n	1	0–1	0	0	0
4	2n	6^c^	0–1	10%	0	0
5	2n	1	0–1	0	0	0
6	2n					
	4n	7	0–1, 7–8	33%	24%	3.9%^d^
7	2n					
	3n					
	4n	5	1, 5–20	46%	20%	3.2%^d^
8A	2n	1	0–1	0	0	0
8B	2n	4	0–2, 3–7	38%	13%	1.9%
	4n					

a Measured in cells of the lowest ploidy level.

b Proportion of cells with dicentric chromosomes, telomeric fusions, or ring chromosomes.

c Clones distinguished mainly by copy-number variability.

d Multiple centrosomes detected by immunofluorescence.

; Figure 2B and C). In many of these cells, nonclonal rings, dicentric chromosomes, or telomeric fusions could be observed by DAPI staining (Figure 2D). Further scoring of G-banded metaphase preparations showed that the tumours with complex karyotypes exhibited these types of nonclonal structural changes in 33–46% of cells (Figure 2E), whereas they were not detected in the other cases, with the exception of a small clonal ring chromosome in case 4. This ring showed little structural variability among different cells, whereas the rings and dicentrics in the complex karyotypes exhibited large intercellular variability in structure and number. Analysis of cell division morphology showed an abnormally high frequency of chromosome bridges (13–24%) compared to fibroblasts (<2%) only in the complex cases (Figure 2F). These tumours also showed multipolar mitoses in a small number of cells. In two of these cases (6 and 7), immunofluorescence staining revealed an elevated number of centrosomes (>2) in approximately 10% of cells, whereas all the other tumours and the fibroblasts showed only one or two centrosomes (Figure 2G and H).

## DISCUSSION

In contrast to the cytogenetic scenario observed in many other epithelial malignant tumours, evidence of clonal evolution has not been presented in RCC (Mitelman *et al*, 2003). Indeed, in this study, four of the cases exhibited no cytogenetic intratumour heterogeneity and one case showed variability limited to chromosome copy number. Taken together, these five tumours contained many of the changes described as primary cytogenetic abnormalities in RCC, including loss of 3p material and polysomies for chromosomes 7, 12 and 17. Analysis of telomere status and mitotic morphology did not show any abnormalities in these cases, dicentric chromosomes were not found, and the ring chromosome observed in one of the cases underwent no further evolution. Interphase FISH analysis did not reveal a higher heterogeneity in chromosome copy number compared to cultured fibroblasts, indicating that the generation rate of numerical chromosome changes was comparable to that in normal cells *in vitro*. This is consistent with statistical analysis showing that the majority of chromosomal changes in RCC most likely depend on rare and mutually independent events (Höglund *et al*, 2001). The three tumours with complex karyotypes (6, 7 and 8B) all exhibited considerable cytogenetic intratumour variability with respect to both structural and numerical changes. In addition, nonclonal rings and dicentrics were common and interphase FISH indicated considerable heterogeneity also regarding numerical changes. In case 8, the two biopsies exhibited highly disparate levels of cytogenetic complexity. All changes found in the simpler karyotype of biopsy A were also seen in the karyotype of biopsy B, indicating that clonal evolution had indeed occurred in a subpopulation of the tumour cells. In all the three cases, signals for telomeric repeats were absent from several chromosome termini, and chromosome bridges were found at anaphase, indicating that the cytogenetic evolution was associated with, if not dependent on, BFB events.

BFB instability has been found in many solid tumours, including osteosarcoma, pancreatic carcinoma (Gisselsson *et al*, 2001), soft tissue sarcoma (Gisselsson *et al*, 2000) and head and neck tumours (Saunders *et al*, 2000). In these neoplasms, evidence of BFB events has been found in the majority of analysed tumours, most of which have shown complex karyotypes. On the other hand, BFB instability has also been found in a small number of borderline malignant or benign lesions with few clonal changes, such as desmoid tumour, pleomorphic adenoma of the salivary gland, and atypical lipomatous tumours (Gisselsson *et al*, 2000, 2002). In the present study, two of the three cases with BFB instability were classified as clear cell and one as papillary carcinoma at histopathological examination. Two of the BFB-positive tumours were locally invasive (pT3a), but also tumour 4 showed an invasive phenotype (pT3c) and patient 5 had developed metastases at diagnosis. Furthermore, the presence of BFB events in RCC showed a correlation neither to patient age, nor to tumour size (data not shown). Hence, BFB instability may occur in both of the main histological classes of RCC and could also be associated with locally aggressive behaviour. Several studies, including highly sophisticated animal models, have suggested that neoplastic cell populations with short telomeres are dependent on the activation of telomerase in order to maintain telomere repeat length and continuing tumour cell proliferation (Greider, 1996; Artandi and DePinho, 2000; Rudolph *et al*, 2001). In tumours not expressing telomerase, alternative lengthening of telomeres through recombination events such as telomere capture may play an equivalent role (Meltzer *et al*, 1993). It is possible that the tumours with BFB instability in the present study had lower levels of telomerase activity compared to those with stable chromosome complements. On the other hand, some studies of pancreatic carcinomas and head and neck carcinomas have demonstrated that critically short telomeres and BFB instability may occur also in the presence of hTERT expression (Gisselsson *et al*, 2001, 2002), indicating that telomerase expression does not completely protect chromosome ends from recombination. Regrettably, quantification of telomerase activity was not possible in the present study due to lack of tumour material.

All the three cases with BFB instability exhibited multipolar cell divisions. This is a common phenomenon in malignant tumours, which may be associated with abnormalities in the configuration of centrosomes (Lingle *et al*, 1998, 2002). Indeed, increased numbers of centrosomes were detected by immunofluorescence in two of the RCC cases with multipolar mitoses. The mechanisms behind the generation of supernumerary centrosomes are poorly understood. The phenomenon has been associated with inactivation of several tumour-suppressor proteins, and overexpression of genes involved in cell cycle regulation, such as the human papilloma virus genes E6 and E7 (Duensing *et al*, 2000) and the human Aurora A gene (Zhou *et al*, 1998). It is possible that the same basic defect in cell cycle regulation might lead simultaneously to BFB instability and mitotic multipolarity. For instance, TP53 dysfunction has previously been associated with the accumulation of supernumerary centrosomes (Carroll *et al*, 1999), and is also known to facilitate the development of unbalanced translocations and epithelial carcinomas through defective telomeres in a murine model (Artandi *et al*, 2000). On the other hand, recent data from an *in vitro* model system overexpressing Aurora A indicates that the generation of supernumerary centrosomes could be dependent on mitotic failure, leading to a duplication of the chromosome complement as well as the number of centrosomes (Meraldi *et al*, 2002). One possible cause of such mitotic failure is anaphase bridges, which may remain unbroken and thus mechanically prevent cytokinesis (McClintock, 1938). In fact, both these mechanisms could explain the positive, linear correlation between the frequencies of anaphase bridges and multipolar mitoses previously found in some genetically unstable tumours (Gisselsson *et al*, 2002). Also, the present study lends support to an association between BFB instability and mitotic multipolarity, as polyploidisation and multipolar cell divisions were restricted to the three RCC cases showing anaphase bridges and telomere dysfunction. Notably, one tumour (case 4) exhibited extensive cytogenetic heterogeneity, but showed neither BFB events nor mitotic multipolarity. In contrast to the cases with complex karyotypes and several related clones, this tumour had both related and unrelated clones, with relatively few and mostly numerical changes. It is possible that this cytogenetic pattern reflects an additional type of chromosome instability, which is largely independent of gross mitotic disturbances.

Taken together, our data suggest the presence of at least two, possibly related modes of cytogenetic evolution in renal cell carcinomas, one occurring through BFB events and the other through multipolar cell division. Both these processes could evolve secondary to a phase in which chromosome mutation is limited to discrete structural and numerical changes, including those that have been described as primary aberrations. When a tumour cell population containing these early changes reaches a critically short telomere length, a second wave of cytogenetic evolution might ensue through mitotic multipolarity and BFB events, leading to complex structural changes and shifts in ploidy level. Alternatively, mitotic instability could occur synchronously to the primary changes in a subgroup of tumours – possibly originating from epithelial cells with unusually short telomeres. Irrespective of the order of events, the molecular background of these largely mechanical chromosomal processes should be an interesting target for future analyses.
